# Japan's Slow Response to Improve Access to Inpatient Care for COVID-19 Patients

**DOI:** 10.3389/fpubh.2021.791182

**Published:** 2022-01-24

**Authors:** Shinji Nakahara, Haruhiko Inada, Masao Ichikawa, Jun Tomio

**Affiliations:** ^1^Graduate School of Health Innovation, Kanagawa University of Human Services, Kawasaki, Japan; ^2^Department of International Health, Johns Hopkins Bloomberg School of Public Health, Baltimore, MD, United States; ^3^Department of Global Public Health, Faculty of Medicine, University of Tsukuba, Tsukuba, Japan; ^4^Department of Public Health, Graduate School of Medicine, The University of Tokyo, Tokyo, Japan; ^5^Department of Health Crisis Management, National Institute of Public Health, Wako, Japan

**Keywords:** disaster medicine, private sector, preparedness, pandemic (COVID-19), healthcare system

## Abstract

The coronavirus disease 2019 (COVID-19) pandemic has exposed various weaknesses in national healthcare systems across the globe. In Japan, this includes the inability to promptly mobilize the resources needed to provide inpatient care in response to the rapidly increasing number of patients. Combined with unclear entry points to healthcare, particularly in emergency cases, this has led to a situation in which access to healthcare is rapidly deteriorating. This study examined problems in Japan's healthcare delivery system. While Japan's healthcare resources (e.g., hospital beds and medical personnel) are comparable to those found in other high-income countries, progress has been slow in securing beds for COVID-19 patients. In addition, the number of beds has only recently reached the levels seen in Western countries. Factors related to slow resource allocation include dispersed existing medical resources (mainly in the private sector), the lack of collaboration mechanisms among private-dominant healthcare providers and public health agencies, an inadequate legal framework for resource mobilization, the insufficient quantification of existing resources, and undesignated entry points to healthcare systems. To better prepare for future disasters, including the next wave of COVID-19, Japan urgently needs to restructure its legal framework to promptly mobilize resources, accurately quantify existing resources, introduce coordination mechanisms with functional differentiations among all community stakeholders, and clearly designate entry points to healthcare.

## Introduction

The coronavirus disease 2019 (COVID-19) pandemic has forced countries around the world to respond with the full extent of their respective healthcare abilities, especially to handle surges in the number of patients. This has highlighted various organizational differences between systems. For example, in the United Kingdom (UK), the command-and-control mechanism of its public healthcare system was employed to mobilize existing resources (e.g., reorganizing healthcare provisions by minimizing routine services); meanwhile, the National Health Service contacted private hospitals to secure block-bought hospital beds, thus increasing the overall capacity to treat COVID-19 patients (1). In the United States (US), various partnerships among public agencies and private-dominant healthcare providers have efficiently functioned to handle similar issues (2, 3).

In contrast, Japan's healthcare system only achieved slow progress in reallocating the medical resources needed to deal with the surge of COVID-19 patients (4). This is not because there has been less disease spread in Japan, as the reported number is unreliable due to limited testing, rather, issues have likely arisen due to a combination of structural issues in the healthcare system and insufficient preparedness in effectively appropriating existing resources in cases of emergency. In this regard, reports have shown that an increased number of patients were waiting for hospitalization during the fifth wave of the pandemic in Tokyo, as of August 2021 (5). Consequently, many of these individuals had to receive treatment at home and not a few at-home deaths were reported (4).

Thus, the pandemic has exposed critical weaknesses in Japan's healthcare system, many of which were masked by previous achievements, including one of the best populational health statuses in the world at a relatively low cost (6, 7). The nation must quickly address problems that have become clear due to COVID-19, both to improve the delivery of health services under normal conditions and prepare for other pandemics and disasters that may arise in the future. As such, this paper tries to clarify and discuss specific problems in Japan's healthcare delivery system, particularly as compared to situations in the UK and US.

## Context: Japan's Health System

Japan's healthcare system can be described as an entity with various similarities to the systems employed in the UK and US; in other words, it is somewhere in between these systems. For example, health service facilities are mostly privately owned, as is the case in the US system (Table 1) (8). Leaving healthcare delivery to the private sector may improve physical access to healthcare services, with a larger number of hospitals than both the UK and US at a relatively low cost to the government. On the other hand, health financing is accomplished through a public health insurance system that covers the entire population, as is done in the UK system, although the UK system differs in that it is tax-based. Here, insurance coverage has ensured service provisions at official prices, even in private hospitals, which may substantially reduce the financial barriers to healthcare services (7).

**Table 1:** Hospitals, hospital beds, COVID-19 patients, and bed occupancies in Japan, the UK, and the US.

	**Japan^**a**^**	**UK^**b**^**	**US^**c**^**
Population (millions)	126	66	327
All hospitals (n)	8,300	1,978	6,146
Publicly owned hospitals (n)	1,524	1,978	1,421
Proportion in all hospitals	18.4%	100.0%	23.1%
Total hospital beds (n)	1,620,040	162,723	924,107
Per 1,000 population	12.8	2.4	2.8
Curative beds^d^ (n)	977,048	–	802,927
Per 1,000 population	7.7	–	2.5
Publicly owned beds (n)	442,741	162,723	197,865
Per 1,000 population	3.5	2.4	0.6
Physicians (n)	315,406	203,529	866,316
Per 1,000 population	2.49	2.95	2.64
Nurses (n)^e^	1,487,444	567,803	3,923,300
Per 1,000 population	11.76	8.45	11.79
Physician-to-bed ratio (total hospital beds)^f^	0.2	1.3	0.9
Nurse-to-bed ratio (total hospital beds)	0.6	3.16	2.93
Maximum daily new confirmed cases^g^	23,083	59,829	251,085
Per one million population^g^	183	877	754
Maximum daily tests performed^h^	135,173	1,303,126	1,909,168
Per 1,000 population^h^	1.07	19.11	5.74
Maximum daily hospital bed occupancy^i^	24,488	39,254	133,210
Proportion in curative beds^j^	2.5%	24.1%	16.6%
Per one million population^i^	194	576	400

*Data source: healthcare resource data were obtained from OECD (8); COVID-19 data were obtained from Our World in Data (9), except for Japanese data on hospital occupancy, which were obtained from the Ministry of Health, Labour and Welfare Japan (10).*

*^a^Japanese data on hospitals and beds were available for 2019. Excluding psychiatric hospitals, there were 7,246 hospitals and 1,374,988 hospital beds. The data on physicians and nurses were from 2018, while those for the nurse-to-bed ratio were from 2017.*

*^b^UK data (estimated number) on hospitals was from 2019, while those on hospital beds were from 2020. The data on physicians, nurses, and nurse-to-bed ratios were from 2020 (estimated).*

*^c^US data on hospitals and hospital beds were from 2018. The data for physicians were from 2019, while those for nurses were from 2020, and those for the nurse-to-bed ratio were from 2018.*

*^d^Curative beds in Japan have various functions (beds for the acute phase, recovery phase, and rehabilitation).*

*^e^Number of practicing nurses for Japan and the UK; and that of professionally active nurses for the US.*

*^f^These figures were calculated based on the numbers of physicians and beds (Japanese and the US data were in 2018, and UK data was in 2020).*

*^g^Data were obtained from Our World in Data (9). These are 7-day rolling averages. The data were from August 25, 2021 in Japan, January 9, 2021 in the UK, and January 8, 2021 in the US.*

*^h^Data were obtained from Our World in Data (9). These are 7-day rolling averages. The data were for August 30, 2021 in Japan, March 21, 2021 in the UK, and November 25, 2020 in the US.*

*^i^The data are from September 1, 2021 in Japan, January 18, 2021 in the UK, and January 14, 2021 in the US. The Japanese data were obtained from the Ministry of Health, Labour, and Welfare (10). UK and US data were obtained from Our World in Data (9).*

*^j^The denominator for the UK data is the number of total hospital beds, assuming that the beds in the UK are used for acute phase curative purposes*.

Including the number of physicians and nurses per capita, Japan's healthcare resources are comparable to those found in the UK and US; even further, the number of hospital beds per capita is much higher than in the UK and US (Table 1). This relatively large number of hospital beds is likely the result of underdeveloped role differentiation for inpatient beds. Physician-to-bed and nurse-to-bed ratios are quite low in Japan, showing that a large proportion of beds are used for long-term care, which requires fewer human resources than acute care. The actual number of acute care (curative) beds should be smaller than that shown in Table 1, but this information is not available in the statistical data.

Compared to the UK and US systems, the most obvious difference in Japan is the lack of gatekeepers. The Japanese government has not assigned gatekeeper roles to primary physicians, meaning that patients can freely choose which medical facilities they wish to visit (i.e., a “free-access” system) (7, 11). Consequently, role differentiations have not emerged between hospitals and clinics; hospitals provide both ambulatory and hospitalized care, although the government has recently begun to introduce role differentiations (11). Among other factors, the insurance system, free access, and large numbers of hospitals and beds have created easy access to hospitalized care.

## Access to Hospitalized Care For COVID-19 Patients

Japan's healthcare system could not promptly meet the demands for hospitalized care that emerged following the patient upsurge created by the pandemic. As of September 1, 2021, reports from the fifth wave in Japan (August to September 2021) showed that the per capita number of maximum daily cases was only around 20% of what was reported in the UK (Table 1) (9, 10). However, this figure may be considerably underreported given the extremely small number of administered tests. From the same time period, Japan's maximum per capita hospital bed occupancy rate was only one-third of that shown in the UK and one-half of that shown in the US. This may reflect insufficient bed allocations rather than lower demands for hospitalized care, especially given the long waiting lists for hospital admission. Compared to numbers from the UK and US, a far lower proportion of curative beds (only 2.5%) were allocated to COVID-19 patients, although not all of these curative beds were actual acute care beds, as mentioned above. Overall, access to hospitalized care has worsened in Japan.

While gradual progress is being made in securing beds for COVID-19 patients in high-demand areas, the rate of improvement is too slow, as the number of available beds was still insufficient. In Tokyo, the maximum hospital bed occupancy was 4,218 as of September 1, 2021 (303 per million persons), which is slightly closer to the US level. However, given that more than 10,000 patients were on the waiting list for hospitalization in August (5), the actual bed requirement at that time was presumably two to three times higher (600–900 per million persons), which is somewhat higher than the maximum level in the UK (576 per million persons). Efforts have since continued to increase the number of secured beds in Tokyo, eventually reaching 6,583 (474 per million persons) as of September 8, 2021; thus matching the level in the US (10).

In terms of hospital access, a unique problem was also revealed in Japan's ambulance system. That is, no hospital emergency departments are designated as ambulance destinations. Rather, ambulance crews perform triage at the scene, then select an appropriate hospital based on the patient's conditions. Upon selection, crews must determine whether their patients will be accepted by sending an inquiry to the hospital (12), which may decline the patient based on its treatment abilities and bed vacancies. In the pandemic context, the number of cases requiring long inquiry processes greatly increased (12, 13). While emergency departments in other countries tended to experience overcrowding when large numbers of COVID-19 patients were transported by ambulance, patients in Japan had to remain at the scene for long periods of time while ambulance crews looked for appropriate hospital destinations. Due to the insufficient number of beds available for treating COVID-19, patients confirmed or suspected of having COVID-19 may thus wait for hours in an ambulance while still in front of their homes (14, 15).

## Identifying the Problems

Several characteristic features of Japan's healthcare system have complicated the ability to allocate resources and secure hospital access during the pandemic. There are five main areas of concern. First, the composition of mostly small- to medium-sized hospitals has made efficient resource reallocation difficult; here, resources are dispersed among these hospitals across communities. More than 80% of private and 50% of public hospitals contain <200 beds (Table 2) (16). Further, these hospitals do not employ experts in infectious disease or contain negative pressure rooms. In addition, approximately 40% of the beds in hospitals containing <200 are used for long-term care (16). As evident from these data and the overall small staff-to-bed ratios (smaller hospitals tend to have smaller ratios), the role of these hospitals is to provide sub-acute to long-term care: they just cannot deal with COVID-19 patients. Even with some abilities to provide acute care, directors of these hospitals may have been reluctant to accept COVID-19 patients by because of concerns about their insufficient ability to manage severe cases, the possible reduction of services to other diseases, and the risk of nosocomial infection from hospitalized COVID-19 patients. A survey conducted by the Ministry of Health, Labour and Welfare in January 2021 indicated that only 19.3% of private hospitals with acute care beds and <200 total beds had hospitalized COVID-19 patients (17).

**Table 2:** Size of medical care facilities and bed types by ownership in Japan (2019).

	**National hospitals^**a**^**	**Public hospitals^**b**^**	**Private hospitals^**c**^**	**Clinics^**d**^**
**Number of hospitals/clinics by hospital size** ^**e**^				
Total^f^	319	1,162	5,765	102,616
0 bed (n)	–	–	–	95,972
Proportion	–	–	–	93.5%
1–19 beds (n)	–	–	–	6,644
Proportion	–	–	–	6.5%
20–99 beds (n)	14	298	2,586	–
Proportion	4.4%	25.6%	44.9%	–
100–199 beds (n)	50	284	2,051	–
Proportion	15.7%	24.4%	35.6%	–
200–399 beds (n)	113	310	855	–
Proportion	35.4%	26.7%	14.8%	–
400 or more beds (n)	142	270	273	–
Proportion	44.5%	23.2%	4.7%	–
**Number of beds by bed type**				
Total^f^	125,533	301,461	857,169	90,825
General curative^g^ (n)	116,886	273,222	497,739	82,943
Proportion	93.1%	90.6%	58.1%	91.3%
Tuberculosis (n)	1,773	1,635	962	–
Proportion	1.4%	0.5%	0.1%	–
Infectious disease (n)	169	1,515	204	–
Proportion	0.1%	0.5%	0.0%	–
Long-term care (n)	380	15,829	292,235	7,882
Proportion	0.3%	5.3%	34.1%	8.7%
Psychiatric (n)	6,325	9,260	66,029	–
Proportion	5.0%	3.1%	7.7%	–

*The data source was a medical facility survey conducted in 2019 by the Ministry of Health, Labour and Welfare (16).*

*^a^This category includes not only hospitals directly affiliated with the Japanese government, but also those affiliated with independent administrative agencies under the jurisdiction of the national government and affiliated with national universities.*

*^b^This category includes local governments, health insurance organizations in the public sector, and public service organizations such as the Red Cross Society.*

*^c^This category includes non-profit-oriented medical corporations and health insurance organizations in the private sector.*

*^d^Private sectors account for 96.0% of all clinics.*

*^e^Hospitals are defined as medical care facilities with 20 or more beds. Clinics can be equipped with fewer than 20 beds. Psychiatric hospitals were excluded.*

*^f^Psychiatric hospitals were excluded.*

*^g^General curative beds are the remainder after excluding specialized beds (beds for infectious diseases, tuberculosis, long-term care, and psychiatric diseases); they are generally categorized as hospital beds intended for various functions (beds for acute phase, recovery phase, and rehabilitation)*.

Second, Japan's system is private-dominant and the government cannot forcibly mobilize medical resources. Most hospitals (79%) and clinics (96%) are owned by entities in the private sector (Table 2). Despite public subsidies, the practice of hospitalizing COVID-19 patients resulted in large revenue losses for many hospitals, which is a strong disincentive among independently financed private hospitals with small budgets (18). In addition, most national hospitals belong to independent external agencies; the majority of public hospitals are either small- to medium-sized or owned by independent agencies or public service organizations such as the Japan Red Cross Society and Japan Agricultural Cooperatives. Neither the national nor local governments have the legal authority to issue orders to hospitals that do not belong to them, and must instead rely on requests. As an exception, prefectural governors hold the legal authority to mobilize resources during major disasters, as outlined in the Disaster Relief Law. However, they cannot exercise this mechanism in the context of COVID-19, as a pandemic is not legally defined as a disaster.

Third, the lack of coordination mechanisms and partnerships among healthcare providers, public agencies, and local governments impedes efficient resource utilization during public health emergencies. Japan's hospital system has traditionally consisted of small- to medium-sized hospitals that developed from clinics and provides self-contained services on an independent basis (7). As such, functional differentiation and coordination are still relatively nascent. Moreover, Japanese municipalities are primarily responsible for disaster responses, including medical care provisions, and must develop community disaster plans under the guidance of prefectural governments (19). However, there are currently no well-developed collaboration or coordination mechanisms between municipal governments and healthcare facilities at the community level; in many cases, there are no clear role definitions or command systems for dealing with patient surges (20). In addition, each municipality or prefecture is responsible for medical care in its jurisdiction, and inter-jurisdiction cooperation mechanisms do not exist (such mechanisms as wide-area patient transfer will be triggered in disasters but a pandemic is not legally defined as a disaster). Consequently, delays may occur when attempting inter-hospital or inter-jurisdiction transfers of COVID-19 patients. Particularly, delayed transfer of recovering patients from tertiary to secondary care hospitals resulted in mismatches between patient severity and hospital function, thus exacerbating the existing supply-demand imbalance in inpatient care.

Fourth, the lack of clearly designated entry points to the healthcare system has diminished access during the pandemic, which is the flip side to the “free access” system. Whereas, there are no strict gatekeeper roles allowing patients to visit any facility they wish (11), patients are required to select appropriate facilities due to premature functional differentiation and the referral network. At the beginning of the pandemic, many hospitals and clinics declined febrile patients because their facilities were not appropriate entry points, meaning that patients should have instead chosen locations that better fit their needs (21). Although conditions are improving, many hospitals continue to decline febrile patients who are being transferred by ambulance, especially during surges of COVID-19 patients (5, 13). In such cases, the patients themselves or ambulance crews must identify facilities that will provide care to febrile patients. In general, the “free-access” provision may entail far fewer entry points to the healthcare system (at the discretion of providers) during public health emergencies, thus creating a situation of “concealed access.” As such, service users may find themselves desperately searching for appropriate entry points (12).

Fifth, medical resources have not been accurately quantified, which may result in incongruent policymaking. At 7.7 per 1,000 persons, Japan appears to have more curative beds per capita than other countries. In reality, this may be an overestimation of Japan's total healthcare resources. Here, curative beds are those which remain after excluding beds for other purposes (infectious diseases, tuberculosis, psychiatry, and long-term care), meaning beds for general usage (11); some are not used for acute care, but are actually taken for rehabilitation or sub-acute care. The number of curative beds designated for acute phase patients should be smaller; a recent study estimated that there were actually 3.3 per 1,000 persons, which may be more accurate (18). The official statistics are likely inaccurate due to the insufficient classification of beds. This has created a seemingly contradictory situation in which a small number of beds are secured for COVID-19 patients despite a large overall number of hospital beds. Furthermore, mobilizing acute care beds for hospitalizing COVID-19 patients resulted in minimizing or postponing other health services, which may mean not only a lack of coordination, but also an absolute lack of resources to deal with a public health emergency while continuing regular services. These problems point to the urgent need for accurate resource assessments.

## Discussion: Propositions For Improvement

Japan is gradually securing the number of hospital beds needed to treat COVID-19 patients. Some areas have even matched the conditions achieved in the US and UK, with most of these being in urban locations. In addition, laws related to infectious disease control were amended in February 2021, such that prefectural governors now have the authority to strongly request that hospitals provide additional beds to accommodate COVID-19 patients. A telemedicine system was also introduced to support small- to medium-sized hospitals that do not employ experts in infectious disease and intensive care to provide appropriate care to COVID-19 patients and to control nosocomial infections. Following another legal amendment designed to facilitate the process, some prefectures have either already set up temporary COVID-19 hospitals or plan to do so in order to increase the number of available beds. Despite these efforts, Japan must still address the problem of inefficient resource usage, by restructuring the medical system to establish better disaster preparedness. Even if it is not currently possible to fundamentally alter the health service system itself (e.g., transitioning from private-dominant to public systems), some useful modifications are feasible. For example, policymakers may incorporate coordination mechanisms, strengthen referral networks, and clearly designate entry points to critical health systems during public health emergencies.

With a private-dominant healthcare delivery system similar to Japan's, the US may serve as a point of reference. Considering the different healthcare systems, we should learn from the coordination mechanisms rather than the system itself. Of note, the US has developed “healthcare coalitions” to coordinate between individual healthcare providers, public health agencies, emergency medical services, and emergency management agencies for health system preparedness. This is supported by the Hospital Preparedness Program, which is a federally funded nationwide initiative (2, 22) that facilitates collaboration among individual healthcare facilities, improves information sharing, and ensures the efficient reallocation of scarce resources in response to all types of public health emergencies. As such, this coordination mechanism among various stakeholders functions as a substitute for an organizational structure with command-and-control mechanisms, as found in public health service systems such as that employed in the UK. The US system may provide a suitable template for improving Japan's health service system.

Although Japanese and US healthcare providers share the similarity of private-dominance, we must pay attention to the differences in referring to the US. The biggest difference is the complexity of provider composition in Japan, in which there is a large number of small- to medium-sized hospitals that independently provide services and sometimes compete with one other in contrast to clear role differentiations among healthcare facilities in the US. Moreover, there are substantial regional variations in this composition. Each region should therefore create an applicable system through a bottom-up approach that involves support from local public health authorities (20). Combined with such region-specific disaster preparedness efforts, the coordination mechanism and governmental subsidies seen in the US healthcare coalitions would expedite functional differentiation and augment the development of inter-hospital resource reallocation mechanisms.

Further, existing laws should be amended to redefine pandemics as “disasters.” This would enable national and local governments to respond more appropriately to pandemics. Although both types of government have the authority to mobilize resources in disasters even from the private sector, the relevant mechanisms cannot be exercised in the context of COVID-19 pandemic, which is not legally defined as a disaster. In the absence of this, management is based on laws that are related to infectious disease control, which give governments the power to make strong requests at most, even after the recent amendment strengthening gubernatorial power. In addition, without full activation of disaster responses, inter-jurisdiction collaboration mechanisms, particularly wide-area patient transfer across prefectural or municipal borders, would not function appropriately. An all-hazards approach would be preferable, such that we can prepare for and respond to all kinds of public health emergencies under the disaster management framework (22).

As mentioned throughout the paper, Japan must designate clear entry points to the healthcare system, with established gatekeeper roles. At the very least, this should be done during public health emergencies, especially in cases with surging numbers of patients. We must absolutely alter the current approach to emergency medical services, in which ambulance crews are tasked with searching for entry points (13–15). Given that medical resources are dispersed across a large number of small- and medium-sized hospitals, there are significant variations in the abilities of individual emergency departments. This highlights the need for a system in which an initial treatment center is available for emergency patients, specifically to accept all patients from a geographically defined area, then redistribute them to appropriate hospitals based on both their own medical conditions and the abilities of the intended facility. This will also facilitate daily operations in general. Even before the pandemic, there were several cases in which ambulance teams had difficulty finding hospitals for their patients. While issues must be addressed in both situations, it is better to overcrowd emergency departments than force ambulances to park throughout the communities.

Accurate quantification of the existing healthcare resources is crucial, especially for use in public health emergencies. However, current figures do not accurately indicate the number of beds available for acute care due to the insufficient differentiation of bed functions in Japan. The Ministry of Health, Labour and Welfare has initiated a system for reporting bed functions to help facilitate this issue, but it is also necessary to reflect differentiations in the official statistics, thus more precisely indicating what resources are available during times of disaster. To obtain information on the available resources in real-time, further utilization of information technology is desirable. Although the Ministry of Health, Labour and Welfare operates a system to collect medical resource information from hospitals throughout the country, its requirement of human data inputting makes collecting information in real-time impossible. A more sophisticated system that can automatically collect information (e.g., using artificial intelligence) would expedite information sharing about available resources (artificial intelligence may also be useful in predicting resource needs). In addition, information sharing mechanisms may also promote local or regional coordination (23).

Finally, we may need to reconsider Japan's regulatory and efficiency-oriented policies to overly streamline its healthcare resources. Seemingly, Japan's current medical resources are insufficient to deal with a public health emergency while continuing regular services reflecting previous efficiency policies to cut the resources to the minimum to maintain regular services. In addition, flexibly increasing hospital beds is not legally allowed. The Ministry of Health, Labour and Welfare has planned to further cut acute care beds with low utilization by reorganizing acute care public hospitals (24). However, in case of emergencies, further resource cuts may need to be reconsidered (to create resource surplus in ordinary times, if not excessive) and flexibility should be introduced to rapidly increase temporary beds.

## Conclusions

Japan's healthcare system has not been able to sufficiently or promptly address a rapid increase in the number of patients who require hospitalization due to COVID-19. Contributing factors include the dispersed nature of existing medical resources, the lack of collaboration mechanisms among private-dominant healthcare providers and public health agencies, an inadequate legal framework for responding to all kinds of emergencies, the insufficient quantification of existing resources, and a lack of designated entry points to healthcare systems. To address these issues and prepare for the next wave of COVID-19 and any future disasters, Japan urgently needs to restructure its legal framework to promptly mobilize resources, accurately quantify existing resources, introduce coordination mechanisms with functional differentiations among all community stakeholders, and clearly designate entry points to healthcare.

## Data Availability Statement

The datasets used for this study can be found (8, 10, 16).

## Author Contributions

SN conceived the idea of the paper and drafted the manuscript. HI, MI, and JT contributed to revising the manuscript. All authors approved the final version.

## Funding

This work was supported by JSPS KAKENHI (No. 18K09967) and the Health, Labour and Welfare Policy Research Grants Research on Health Security Control (No. 19LA2003).

## Conflict of Interest

The authors declare that the research was conducted in the absence of any commercial or financial relationships that could be construed as a potential conflict of interest.

## Publisher's Note

All claims expressed in this article are solely those of the authors and do not necessarily represent those of their affiliated organizations, or those of the publisher, the editors and the reviewers. Any product that may be evaluated in this article, or claim that may be made by its manufacturer, is not guaranteed or endorsed by the publisher.
